# Supplementary material for: Case Studies in Pediatric Endocrinology: An Interactive Learning Module
Source: MedEdPORTAL. 2016 Sep 13;12:10456. doi: 10.15766/mep_2374-8265.10456 (PMC6464428; doi:10.15766/mep_2374-8265.10456)
Supplement: Supplementary file 1 — A. Case Studies In Pediatric Endocrinology.ppt B. Survey Data.xls [file mep-12-10456-s001.zip › _Educational Summary Report_10456.pdf]

MedEdPORTAL®
The Journal of Teaching and Learning Resources

Crossref

Original Publication

OPEN ACCESS

Case Studies in Pediatric Endocrinology: An Interactive Learning Module

Biva Narsing*, Daina Dreimane, MD
*Corresponding author: bivanarsing@gmail.com

Citation: Narsing B, Dreimane D. Case studies in pediatric endocrinology: an interactive learning module. *MedEdPORTAL.* 2016;12:10456. https://doi.org/10.15766/mep_2374-8265.10456

Copyright: © 2016 Narsing and Dreimane. This is an open-access article distributed under the terms of the Creative Commons Attribution license.

Appendices

A. Case Studies In Pediatric Endocrinology.ppt
B. Survey Data.xls

All appendices are peer reviewed as integral parts of the Original Publication.

Abstract

Introduction: Pediatrics is an exciting field because it requires having a background in physiology that evolves as the patient ages. As a subspecialty, pediatric endocrinology encompasses a wide range of disease processes both acute and chronic. This module was created to provide a review of endocrine physiology, promote understanding of the biopsychosocial model of children diagnosed with an endocrine disorder, and utilize simulated case studies to become familiar with patient management. **Methods:** The material and case studies presented are based on firsthand experiences in a pediatric clinic and diabetes camp, as well as an extensive literature review. The information in this resource teaches interview questions and problem-solving techniques and ensures that the learner understands the basic equipment used by the patients. Implementation of this module will advance students' and residents' efficacy in caring for this pediatric population. The approximate time to complete this module is 3 to 5 hours. To evaluate the effectiveness of this module, pre- and postmodule surveys were administered to medical students. Factors analyzed included overall user satisfaction, utility of the module, comfort in approaching pediatric endocrine patients, and suggestions for improvement. **Results:** A cohort of medical students (*N* = 26) completed both surveys and the module, with an equal distribution of first- and second-year students to third- and fourth-year students. The surveys showed a general trend of improvement in self-reported comfort with both basic science and clinical skills components. **Discussion:** This module would be most beneficial to a medical student who may rotate with a pediatric endocrinologist during the clinical years. The target audience can be broadened to include not only medical students but also physician assistant students, nursing/nurse practitioner students, pediatric residents, and pediatric endocrinology fellows.

Keywords

Type 1 Diabetes Mellitus, Pediatrics, Case Studies, Pediatric Endocrinology, Thyroid Pathology

Educational Objectives

By the end of this session, the learner will be able to:

1. Explain the mechanisms involved in the biosynthesis, storage, and secretion of triiodothyronine (T3) and thyroxine (T4).
2. Explain the regulation of thyroid secretion and the regulatory feedback loop.
3. Describe the actions of thyroid hormones throughout the body.
4. Describe the actions of insulin.
5. Describe the actions of glucagon.
6. Understand how to gather a history from a new endocrine patient effectively.
7. Recognize the generic and brand names of the different types of insulin.
8. Describe the onset of action and duration of action of the different types of insulin
9. Understand how to create insulin regimens and how to adjust insulin dosages based on blood glucose logs.

10. Teach a patient how and where to administer insulin injections.
11. Explain the rationale behind the basal-bolus regimen used in insulin pumps.
12. Understand how to counsel a type 1 diabetic patient on diet and exercise goals.
13. Calculate a mealtime insulin dosage by using a carbohydrate ratio and a correction factor/sliding scale.
14. Evaluate the role that school nurses and teachers play in the overall care of a type 1 diabetic.
15. Integrate information from the history, physical, and pertinent labs in order to establish a diagnosis.
16. Formulate an assessment and plan, along with the necessary follow-up appointments, once a diagnosis has been established.
17. Be aware of the need for family support when diagnosing a patient with a chronic illness.
18. Understand the specific criteria used to diagnose diabetes mellitus.
19. Determine which patients will benefit from the use of an insulin pump and why.
20. Adjust pump settings as necessary based on the patient's pump data.
21. Explain the pathophysiology of hyperthyroidism and determine which labs to order to confirm diagnosis.

Introduction

This module serves as a useful tool to understand the subspecialty of pediatric endocrinology. The compilation of case studies, along with intervening physiology presented in this resource, can supplement a user's pediatric rotation. Exposure to pediatric endocrinology is somewhat limited at most institutions, so this module will be able to give a well-rounded introduction to this field. Common pediatric endocrinology cases, primarily related to thyroid disease and diabetes, are explored within this module. The reason for highlighting these conditions is that they represent a high occurrence of pediatric endocrine cases encountered in clinical settings.

Medical students who are planning on rotating with a pediatric endocrinologist during their clinical years are a subset of the main target audience. Pediatric residents can also benefit from this module because it encompasses the biopsychosocial model of care, including information on how to advise on diet and exercise, how patients manage their medical care at school, and how families cope with a diagnosis of a chronic illness. These principles have the utility to be applied to multiple fields throughout pediatrics. Keeping that in mind, this module can also target students belonging to various branches of the health science field, including nursing, nurse practitioner, physician assistant, and social work students. A prerequisite of an endocrinology course is needed to utilize this resource to its fullest capacity.

In relation to other existing resources that review pediatric endocrinology cases, there are myriad textbooks and journals dedicated to publishing up-to-date research, which we referenced when conducting a literature review to develop this resource.[1-18] A unique aspect of this particular resource is that it does not just present case studies. The interactive approach allows a user to learn the basics of the disease process, make decisions regarding the patient's care, and also think about the long-term outcomes of different treatment plans. This module serves as a convenient and interactive way to review a range of endocrinology cases, as well as other important aspects of patient care.

Methods

Creation of this module was brought about by our participation in the Texas Tech Apprenticeship Program, designed to allow first- and second-year medical students early clinical exposure and contact with faculty. The program consisted of an apprenticeship in a pediatric endocrinology clinic and a role serving as a counselor at a pediatric diabetes summer camp. The material and case studies presented are based on these firsthand experiences, as well as an extensive literature review.

Key aspects of the module's design include self-pacing and user interaction. The presentation (Appendix A) aims to simulate a rotation in a pediatric endocrinology clinic by presenting common topics followed by real-world case studies. A generalized understanding of endocrine disorders is needed to fully utilize this material; however, no additional resources are necessary.

This module was created using Microsoft PowerPoint. By viewing the module in presentation mode, the user is able to navigate through certain subsections, answer questions, and proceed through the rest of the presentation. This presentation can be used by a single user or in a small-group setting or can be presented to a larger group for more of a discussion-based interaction.

Since the Results section below presents the analyzed data, we have also included a file depicting the raw data (Appendix B). This file was made available in case users wish to see the raw data to draw their own conclusions about the efficacy of this resource.

Results

The interactive module was tested by utilizing a focus group of medical students along with pre- and postsurveys. A cohort of medical students (N = 26) completed both surveys and the module, with an equal distribution of first- and second-year students to third- and fourth-year students. At the time of survey initiation, first-year medical students had completed a basic endocrinology physiology course.

Details of the quantitative analysis of the study are shown in the Table. The answer scale used in the surveys was from 1 to 5, with 1 being the least likely/useful and 5 being the most likely/useful. As is evident in the Table, all the p values comparing both the pre- and postsurveys were less than .01. Although it is not appropriate to state that this change is statistically significant based on the small sample size of 28 and no sample-size calculation, a general trend of these data can still illustrate the utility of this module. The general trend shown is that users become more confident in their knowledge base after completing the interactive module and appear to feel more comfortable in clinical skills as well. Users ranked the usefulness of the module as 4.27 (± 0.72) and the overall satisfaction as 4.35 (± 0.69), showing that the large majority of medical students benefitted from this interactive learning module.

Table. Details of Quantitative Analysis of Learning Resource

	Presurvey		Postsurvey		
**Survey Question**	**Average**	***SD***	**Average**	***SD***	***p***
How comfortable are you in approaching a pediatric endocrinology patient in a clinical setting?	2.41	0.966	3.692	0.549	<.01
How would you rate your understanding of the function/physiology of the thyroid gland and endocrine pancreas?	3.462	0.79	4.154	0.464	<.01
How familiar are you with the usage and indications of various types of insulin?	2.718	1.025	3.885	0.816	<.01
Would you feel comfortable implementing a diet and exercise plan for a type 1 diabetes mellitus patient?	0.256	0.442	0.692	0.471	<.01
How likely are you to use an additional resource to learn about pediatric endocrinology?	3.513	1.097			N/A
How useful was this module in learning to approach pediatric endocrinology patients?			4.269	0.724	N/A
What is your overall satisfaction with this learning module?			4.346	0.698	N/A

Blank cells indicate questions that were included in either the pre- or postsurvey, but not in both; p values are available only for those questions included in both the pre- and postsurveys.

Qualitative metrics were also obtained in the form of a question inquiring about suggestions for improvement. One user suggested adding a voice-over to explain certain parts of the presentation. Another user expressed satisfaction in the module with the statement "I would love if one of these was made for each clinical rotation in my future." Overall, the module received very positive results in this focus group.

Discussion

This interactive module was created to accomplish a few goals, namely, to review endocrine physiology, understand the biopsychosocial model of children diagnosed with an endocrine disorder, and utilize simulated case studies to become familiar with patient management. A culmination of personal experiences in a pediatric endocrinology clinic and diabetes summer camp, as well as an extensive

literature review, served as the inspiration for designing the module. When a user walks through the interactive learning module, he or she is able to learn the basic science and clinical skills needed to excel in a pediatric endocrinology rotation. Testing this module using a cohort of medical student peers proved to be very successful. Survey data showed that students were amenable to using a resource outside of their school materials to study for pediatric endocrinology. It is encouraging that this same group of students rated this specific resource highly in both utility and satisfaction. The main target audience includes medical students who will rotate with a pediatric endocrinologist, but pediatric residents would most likely also benefit from this content. As stated in the Introduction, the fact that this module presents extra information in addition to case studies allows the user to have the full range of information available in just one resource. A medical student can learn the basics of a disease process, then attempt to manage a case based on what has been learned. The survey results indicate that users experienced an increase in both basic science and clinical skills components. A medical student or resident at any institution can utilize this resource to become more familiar with the pediatric endocrine population and its specific needs.

A major challenge encountered when developing this module was creating a resource that could supplement traditional medical school education without being too repetitive. The process of categorizing information into logical subcategories made the module less intimidating and easier to comprehend. It was important to include aspects of the biopsychosocial model in this project because the unique challenges of chronic pediatric endocrine patients are not necessarily highlighted or discussed in depth in medical school. After spending a week at a diabetes summer camp, it was interesting to gain insight into exactly what the day-to-day life of children with type 1 diabetes mellitus is truly like. By explaining insulin regiments, insulin pumps, school care, and so on, this module aims to provide a glimpse into a typical day of a type 1 diabetic's life.

One limitation of this module is that it is not formatted to be used on a mobile device. The intended medium was a PowerPoint slide show in presentation mode on a laptop or desktop computer, but feedback from the surveys indicated that the module does work well on tablets. With a huge majority of medical students using a tablet or laptop as their main accessory for studying, this module is largely accessible. A potential future application for this module is for it to be used in a group setting, in which students can work through the module together and discuss content when conflict arises. There is the option for only the case-study portion of the module to be used in this type of a setting.

Biva Narsing: Fourth-year Medical Student, Texas Tech University Health Sciences Center School of Medicine

Daina Dreimane, MD: Assistant Professor of Pediatrics, Texas Tech University Health Sciences Center School of Medicine; Chief, Division of Endocrinology and Diabetes, Texas Tech University Health Sciences Center School of Medicine

Disclosures
None to report.

Funding/Support
None to report.

Ethical Approval
Reported as not applicable.

References

1. Brent GA. Mechanisms of thyroid hormone action. *J Clin Invest.* 2012;122(9):3035-3043. http://dx.doi.org/10.1172/JCI60047
2. Costanzo LS. *Physiology*. 5th ed. Philadelphia, PA: Lippincott Williams & Wilkins; 2011.
3. Fu Z, Gilbert E, Liu D. Regulation of insulin synthesis and secretion and pancreatic beta-cell dysfunction in diabetes. *Curr Diabetes Rev.* 2013;9(1):25-53. http://dx.doi.org/10.2174/157339913804143225
4. Toy EC, Yetman RJ, Girardet RG, et al. *Case Files: Pediatrics.* 3rd ed. New York, NY: McGraw-Hill; 2010.

5. Bangstad H-J, Danne T, Deeb L, Jarosz-Chobot P, Urakami T, Hanas R. Insulin treatment in children and adolescents with diabetes. *Pediatr Diabetes.* 2009;10(suppl 12):82-99. http://dx.doi.org/10.1111/j.1399-5448.2009.00578.x
6. Court JM, Cameron FJ, Berg-Kelly K, Swift PG. Diabetes in adolescence.*Pediatr Diabetes.* 2009:10(suppl 12);185-194. http://dx.doi.org/10.1111/j.1399-5448.2009.00586.x
7. Zib I, Raskin P. Novel insulin analogues and its mitogenic potential. *Diabetes Obes Metab.* 2006;8(6):611-620. http://dx.doi.org/10.1111/j.1463-1326.2005.00567.x
8. Golden SH, Brown T, Yeh HC, et al. *Methods for Insulin Delivery and Glucose Monitoring: Comparative Effectiveness.* Rockville, MD: Agency for Healthcare Research and Quality; 2012. AHRQ publication 12-EHC036-EF.
9. Silverstein J, Klingensmith G, Copeland K, et al. Care of children and adolescents with type 1 diabetes: a statement of the American Diabetes Association. *Diabetes Care.* 2005;28(1):186-212. http://dx.doi.org/10.2337/diacare.28.1.186
10. White RD. Insulin pump therapy (continuous subcutaneous insulin infusion). *Prim Care.* 2007:34(4):845-871. http://dx.doi.org/10.1016/j.pop.2007.07.005
11. Protecting students with disabilities. U.S. Department of Education Web site. http://www2.ed.gov/about/offices/list/ocr/504faq.html. Accessed May 2014.
12. Swift PG. Diabetes education in children and adolescents.*Pediatr Diabetes.* 2009;10(suppl 12):51-57. http://dx.doi.org/10.1111/j.1399-5448.2009.00570.x
13. Corwell B, Knight B, Olivieri L, Willis GC. Current diagnosis and treatment of hyperglycemic emergencies. *Emerg Med Clin North Am.* 2014;32(2):437-452. http://dx.doi.org/10.1016/j.emc.2014.01.004
14. Rosenbloom AL, Silverstein JH, Amemiya S, Zeitler P, Klingensmith GJ. Type 2 diabetes in children and adolescents.*Pediatr Diabetes.* 2009:10(suppl 12):17-32. http://dx.doi.org/10.1111/j.1399-5448.2009.00584.x
15. Butwicka A, Zalepa A, Fendler W, Szadkowska A, Mlynarski W. Maternal depressive symptoms predict acute hospitalization among children with type 1 diabetes. *Pediatr Diabetes.* 2013:14(4):288-294. http://dx.doi.org/10.1111/pedi.12010
16. Delamater AM. Psychological care of children and adolescents with diabetes.*Pediatr Diabetes.* 2009;10(suppl 12):175-184. http://dx.doi.org/10.1111/j.1399-5448.2009.00580.x
17. Amer KS. Advances in assessment, diagnosis, and treatment of hyperthyroidism in children. *J Pediatr Nurs.* 2005;20(2):119-126. http://dx.doi.org/10.1016/j.pedn.2004.12.013
18. Low iodine diet. American Thyroid Association Web site. http://www.thyroid.org/faq-low-iodine-diet/. Accessed October 2014.

Received: March 21, 2016 | **Accepted:** September 2, 2016 | **Published:** September 13, 2016
